# Nutrient supplements boost yeast transformation efficiency

**DOI:** 10.1038/srep35738

**Published:** 2016-10-20

**Authors:** Sheng-Chun Yu, Alexander Dawson, Alyssa C. Henderson, Eloise J. Lockyer, Emily Read, Gayathri Sritharan, Marjah Ryan, Mara Sgroi, Pok M. Ngou, Rosie Woodruff, Ruifeng Zhang, Travis Ren Teen Chia, Yu Liu, Yiyu Xiang, Pietro D. Spanu

**Affiliations:** 1 Department of Life Sciences, South Kensington Campus, Imperial College London, London, SW7 2AZ, United Kingdom

## Abstract

Efficiency of yeast transformation is determined by the rate of yeast endocytosis. The aim of this study was to investigate the effect of introducing amino acids and other nutrients (inositol, adenine, or p-aminobenzoic acid) in the transformation medium to develop a highly efficient yeast transformation protocol. The target of rapamycin complex 1 (TORC1) kinase signalling complex influences the rate of yeast endocytosis. TORC signaling is induced by amino acids in the media. Here, we found that increasing the concentration of amino acids and other nutrients in the growth media lead to an increase yeast transformation efficiency up to 10^7^ CFU per μg plasmid DNA and per 10^8^ cells with a 13.8 kb plasmid DNA. This is over 130 times that of current published methods. This improvement may facilitate more efficient experimentation in which transformation efficiency is critical, such as yeast two-hybrid screening.

*Saccharomyces cerevisiae* transformation efficiency has received much attention in recent years^1^; this is particularly important in the application of yeast two–hybrid screening for protein-protein interactions^2^. In general, yeast transformation efficiency depends on endocytotic membrane invagination and cell wall structure alterations^3^. Endocytosis and cell wall structure alterations can be triggered by biological^4^,^5^,^6^ or physical methods^7^,^8^. However, the transformation efficiencies achieved by these methods are low^1^,^9^.

One way to improve yeast transformation efficiency is to enhance endocytosis, followed by escaping the traditional endosome pathway in *S. cerevisiae*^10^. For example, Schiestl and Gietz modified the monovalent alkali cations/PEG method with inclusion of single-stranded carrier DNA (ss-DNA) to increase plasmid DNA binding to productive endocytotic binding sites^11^.

More recently, the LiAc/ss carrier DNA/PEG method was improved to achieve 1 × 10^6^ transformants per μg plasmid DNA per 10^8^ cells^4^. However, we have found no record of whether presence of amino acids or other nutrients in the media prior to transformation affects transformation efficiency.

In order to improve yeast transformation conditions, we applied response surface methodology (RSM) to infer experimental conditions for optimal transformation efficiency. RSM is widely used to improve industrial processes^12^. Yeast transformation efficiency is affected by heat-shock time and the amount of plasmid. Furthermore, the composition of transformation mix also has an impact. In this study, we investigate some of these factors affecting yeast endocytosis and the effect of adding amino acids and other nutrients (hereafter referred to as nutrient supplements) in the incubation media immediately prior to transformation. This is then followed by RSM analysis to optimise the composition of yeast transformation reagent.

## Results

**Figure 1:**
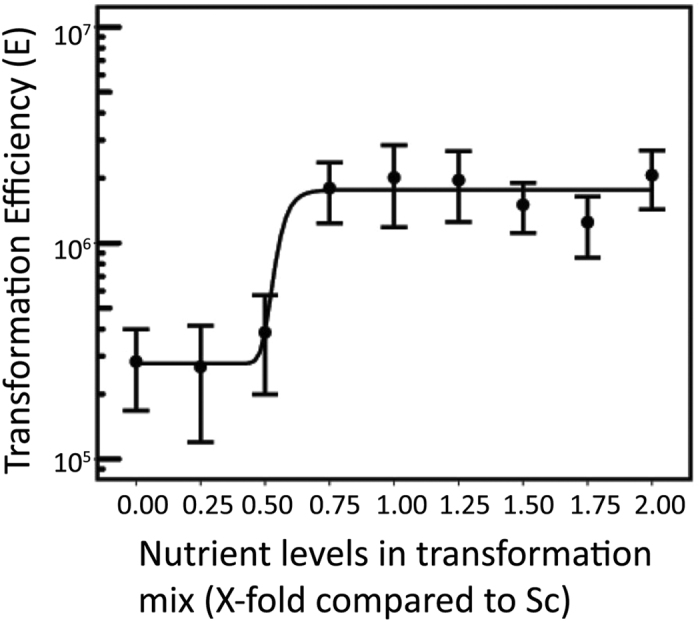
Effect of nutrient supplement concentration in the transformation mix on transformation efficiency. A range of concentrations of nutrient supplements was tested by using the *S. c*. EasyComp™ Transformation kit. The 10x AA mix with the other nutrients was added into solution II and solution III to obtain the final levels in the solution indicated. The transformation reactions were carried out with endotoxin-free plasmid DNA (0.5 μg) and 30 minute heat shock at 37 °C. These values are the means and standard deviation of 5 independent replicates. Transformation Efficiencies are given as colony-forming unit (CFU) per μg plasmid DNA per 10^8^ cells.

The efficiency of yeast transformation in previous research using the LiAc/ss-DNA/PEG protocol indicated that approximately 1 × 10^6^ CFU per μg plasmid DNA per 10^8^ cells can be expected^4^,^13^. The plasmids used to estimate the maximum efficiency were approximately 5 kb^14^,^15^. This does not reflect the size of plasmid DNA commonly used in real applications of yeast transformation. In this study, we used a relatively large plasmid (13.8 kb) to develop a modified Sodium hydroxide-Bicine/LiAc/Poly(ethylene glycol)/ss-carrier DNA/Amino Acids (SuccessAA) protocol. Previous research indicated that the level of intracellular amino acids can enhance yeast endocytosis via TORC1 signalling complex^16^. We therefore tested the effect of adding nutrient supplements to the medium used to prepare competent cells. In order to compare transformation efficiencies to other established protocols, we also added nutrient supplements to the commercially available *S.c.* EasyComp™ Transformation kit (Thermo Fisher Scientific). Figure 1 shows the effect of nutrient supplement addition on yeast transformation efficiency. Firstly, the effect of adding nutrient supplements to the yeast transformation mix was examined (Fig. 1). The transformation was performed with 0.5 μg plasmid DNA and 37 °C heat shock for 30 minutes. Addition of no amino acids mix (No AA) or amino acids mix (AA) with the other nutrients at less than 0.5x of those found in “Synthetic complete” (Sc) medium resulted in transformation efficiencies of approximately 3 × 10^5^ CFU per μg plasmid DNA per 10^8^ cells. When the concentration of nutrient supplements in the transformation mix increased to over 0.75x, the transformation efficiency reached approximately 2 × 10^6^ CFU per μg plasmid DNA per 10^8^cells, which is consistent with results obtained in previous research^16^. The relationship between nutrient supplements concentration in the transformation mix and yeast transformation efficiency can be approximated using a sigmoid function.

In order to further increase the transformation efficiency, we tested the effect of altering two other parameters (time of heat shock and amount of DNA in the transformation mix). We used RSM to determine the optimal levels for transformation of these parameters.

**Figure 2:**
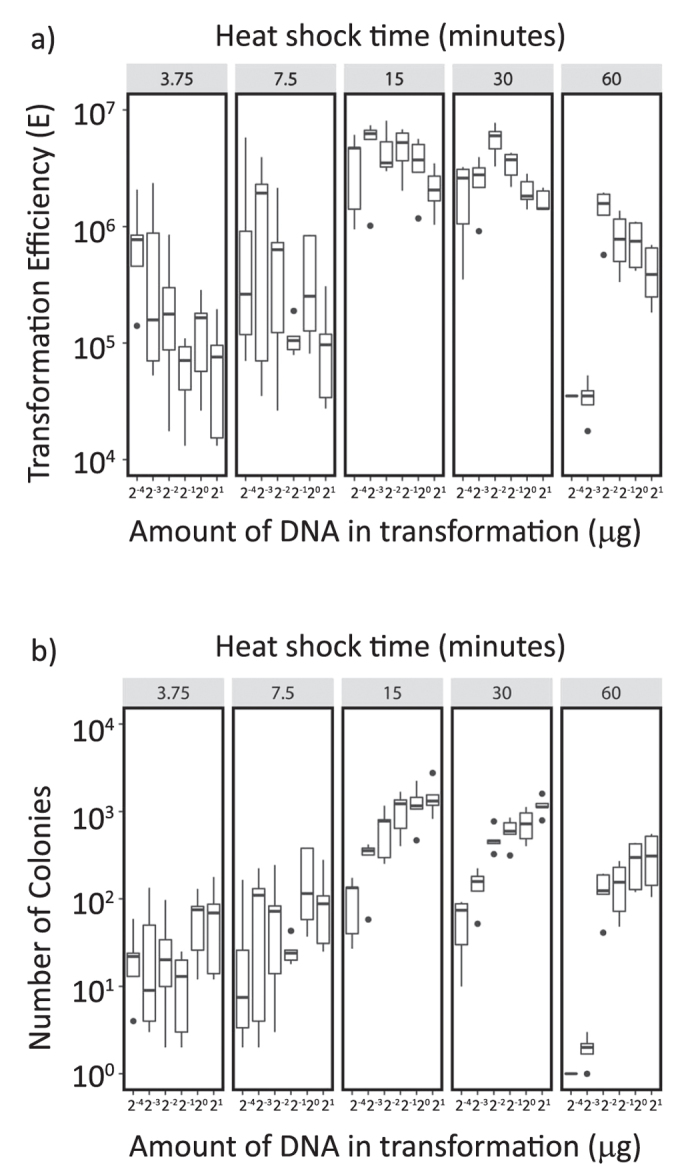
The effect of heat shock time, and the amount of plasmid DNA used in the transformation reactions on Transformation Efficiency. The relationship between (**a**) transformation efficiency or (**b**) number of colonies, the heat shock time, and the amount of plasmid DNA was investigated. Yeast strain MaV203 was transformed using the *S. c*. EasyComp™ Transformation kit. The level of nutrient supplements in the transformation mix and freezing solution was maintained at 1.25x compared to Sc. The amount of plasmid DNA ranged from 0.0625 μg to 2 μg and the time for heat shock ranged from 3.75 minutes to 60 minutes. The interquartile range (IQR), the medians (horizontal bars in IQRs), the outliers and the whiskers of each transformation conditions are shown in these figures. All the experiments were performed 5 times independently.

Transformation efficiencies of 15 and 30 minute heat-shock were significantly different from the others (assessed using generalized linear model). With further two-way ANOVA analysis, we found that the transformation efficiencies of 15 and 30 minute heat shock were significantly different (p = 0.0012). In some conditions, the transformation efficiency of 15 minute heat shock reached approximately 1.2 × 10^7^ CFU per μg plasmid DNA and per 10^8^ cells. In Fig. 2, we show the Transformation Efficiencies and the Number of Colonies obtained varying time of heat shock and amount of DNA in the transformation mixes. The data is displayed as box-and-whiskers plots. The circles in these figures are outliers (as determined by R). The horizontal lines in each bar are the medians of each data set. Also, the bars are inter-quartile ranges (IQRs) which represent the 25th (bottom of the box) and 75th (top of the box) percentiles. The vertical lines above and below each box are the “whiskers” of the boxplot.

For example, the upper whisker in Fig. 2a (the third graph, data for 2^−2^) is the 75th percentile of transformation efficiency plus 1.5xIQR (8 × 10^6^ CFU per μg plasmid DNA and per 10^8^ cells). Based on this, 1.2 × 10^7^ CFU per μg plasmid DNA and per 10^8^ cells is not shown in Fig. 2a. The inter-quartile ranges (IQRs) and the whiskers of 15-minute heat shock in both transformation efficiency and colony count were frequently much wider than those of 30-minute heat shock. Hence, thereafter we focused on the efficiencies of 30 minute heat shock. In this case, we found that the efficiency reached maximum when the heat shock time and plasmid DNA used in the transformation were 30 minutes and 0.25 μg, respectively; efficiency is also significantly different from the other conditions of 30 minute heat shock using one-way ANOVA analysis (p < 0.0001) (Fig. 2a). In this condition, the average number of colonies on a 9 cm diameter Petri dish reached nearly 500 by only plating 10% of the transformation reaction (Fig. 2b). The transformation efficiency and the number of colonies were very variable when the heat shock time was less than 15 minutes; also, the efficiency decrease when the cells were heat shocked for 60 minutes (Fig. 2a,b).

**Figure 3:**
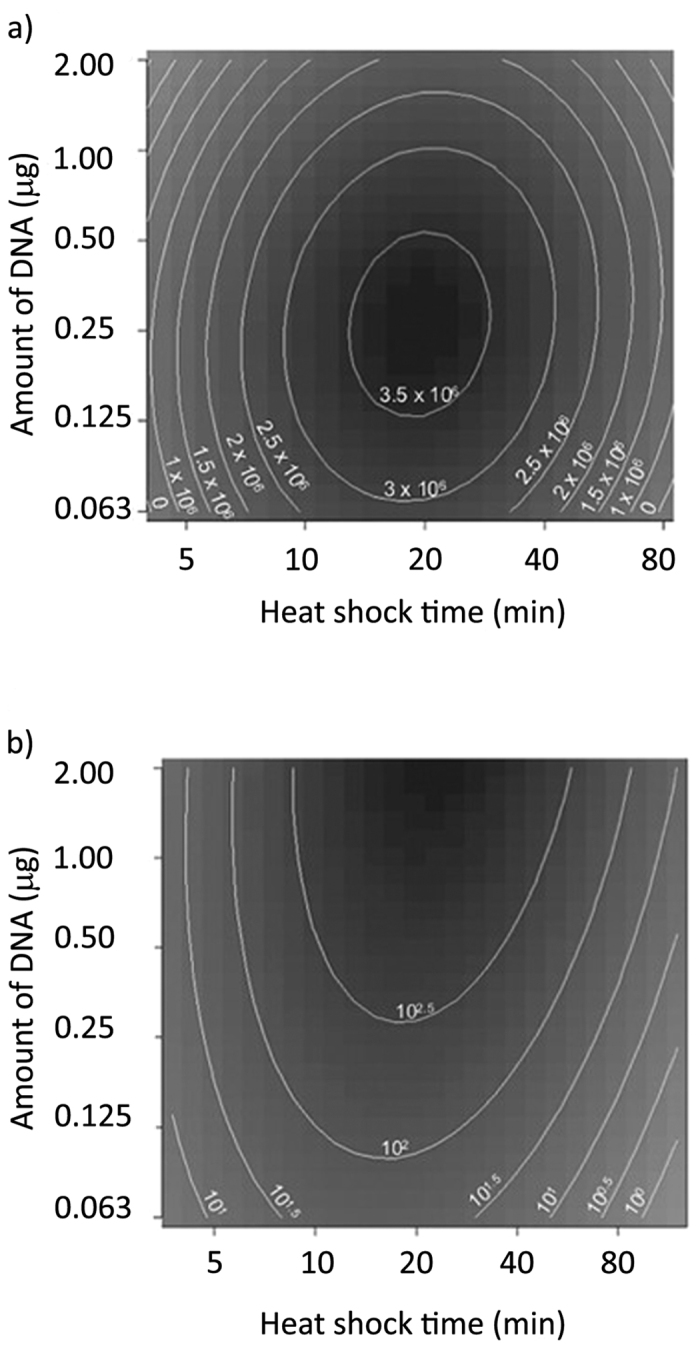
Response Surface Method (RSM) plots to predict the best transformation conditions. The RSM package in R was used to determine optimal **(a)** transformation efficiencies or **(b)** number of colonies obtained under different conditions. These contour plots indicated that the optimal MaV203 transformation conditions occurred in the following ranges; plasmid DNA: 0.2 to 0.3 μg; the heat shock time: 15 minutes to 30 minutes.

RSM analysis was applied to determine the best condition for yeast transformation, based on the experimental efficiency/colony data within the reasonable ranges of transformation factors. Optimal heat-shock time ranged from 15 minutes to 30 minutes and the amount of plasmid DNA ranged from 0.2 μg to 0.3 μg (Fig. 3a,b). Because of the need to generate a sufficiently high absolute number of colonies that allow downstream applications, as well as high efficiency and we suggest that 0.25 μg of plasmid DNA is used in the transformation.

**Figure 4:**
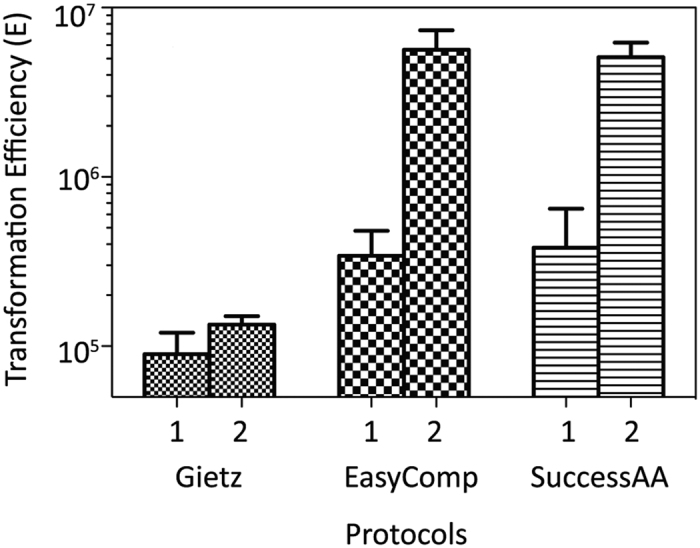
Comparison of efficiencies of transformation between different protocols used. Different protocols were compared with the large (13.8 kb) plasmid DNA. The experiments for each protocol were performed 5 times independently. The conditions were the same as those for the MaV203 transformation but the heat shock time and temperature were 30 minutes and 37 °C, respectively. In each protocol, we tested the effect of nutrient supplement addition. The final levels of nutrient supplements in +AA in the mix was 1.25x; −AA had no nutrient supplements in the mixes.

Finally, we compared directly the effect of nutrient supplement addition on yeast transformation efficiency between Gietz’s protocol, the commercially available *S.c.* EasyComp™ Transformation kit, and our SuccessAA protocol (Fig. 4). Comparing different transformation efficiencies from various research is challenging. The efficiency is influenced by the plasmid size, the number of cells, heat shock time, or even the yeast strain. If different protocols are to be compared, it is necessary to compare protocols under the same conditions and experimental settings. Because on this, all the different protocols in this study were performed with 13.8 kb plasmid. Addition of nutrient supplements to the transformation mixture led to higher efficiencies in all three conditions/methods. Although the mean yeast transformation efficiency of nutrient supplement addition in Gietz’s protocol was marginally higher than without nutrient supplement addition, the difference is not significant (p = 0.1239). Consistently with the previous results, addition of nutrient supplements to the transformation solutions resulted in large, statistically significant increases in transformation efficiencies in both the commercial preparation (EasyComp Kit, Thermo Fisher Scientific) and the formulation described in this paper (a 16- and a 13-fold increase, respectively). It should be noted that there were no statistically significant differences in the efficiencies between the commercial kit and the SuccessAA formulation irrespective of the addition of nutrient supplements. The maximum efficiency reached was about 1.2 × 10^7^ CFU/μg DNAx 10^8^ cells with SuccessAA formulation and with 13.8 kb plasmid. These results indicate that our SuccessAA protocol is a simple and efficient method for yeast transformation, far higher than that of published formulations (Gietz’s protocol; about 63- fold) and about 15-fold that of commercially available kit without nutrient supplement addition.

## Discussion

Published work documented improvements of the LiAc/ss-DNA/PEG reaching transformation efficiencies up to 1 × 10^7^ CFU per μg plasmid DNA and per 10^8^ cells, which may result from dehydrating the membrane and enhancing the permeability of the membrane to Ca^2+^ and other ions^3^. However, those studies used considerably smaller plasmids (plasmid YCplac33 (5603 bp) and plasmid YEplac 195 (5241 bp)). Using our method, we obtained and exceeded these efficiencies using a 13.8 kb plasmid that is commonly used in practical applications in yeast methods such as Y2H.

In this study, we tested the effect of nutrient supplements in the transformation media. These are important factors influencing rates of yeast endocytosis via the TORC1 signalling pathway. Comparing the highest efficiency of our SuccessAA protocol with the lowest efficiency of Gietz’s protocol with our experimental conditions, we found that addition of nutrient supplements boosted transformation efficiency up to 200-fold. These findings are in agreement with observations that yeast transformation relates to endocytosis-like processes of exogenous DNA^3^. This improvement by nutrient supplements is simple and easy to achieve in any lab or any industrial environment. It is plausible that the improvement is due to a synergistic effect which comes from all or some of the nutrients including amino acids, inositol, adenine, and p-aminobenzoic acid. Discovering the “real” players to contribute to the transformation efficiency improvement would be the future work of this study. Some limitations are worth noting. Although nutrient supplements can improve the transformation efficiency significantly, they have to be prepared freshly due to their nature of sensitivity to light and temperature and due to the decay with time. Nevertheless, our results represent a feasible and novel high transformation efficiency protocol for *S. cerevisiae*.

## Methods

### Reagents and equipment

*S. c*. EasyComp™ Transformation kit (K5050-01, ThermoFisher Scientific), Yeast extract (Y1625-250G, Sigma-Aldrich), Peptone (P5905-1KG, Sigma-Aldrich), Adenine hemisulfate salt (A3159-100G, Sigma-Aldrich), D-(+)-Glucose (G7021-1KG, Sigma-Aldrich), yeast nitrogen base without amino acids (Y0626-250G, Sigma-Aldrich), yeast synthetic drop-out medium supplements without histidine, leucine, tryptophan, and uracil (Y2001-20G, Sigma- Aldrich), L-histidine monohydrochloride monohydrate (53370-100G, Sigma-Aldrich), L- tryptophan (T8941-25G, Sigma-Aldrich), uracil (U1128-25G, Sigma-Aldrich), D-sorbitol (S3889-1KG, Sigma-Aldrich), Bicine (B3876-100G, Sigma-Aldrich), LiAc(6108-17-4, Alfa Aesar), ethylene glycol(324558-100ML, Sigma-Aldrich), dimethyl sulfoxide (DMSO) (D2650-5 × 5ML, Sigma-Aldrich), Poly(ethylene glycol) BioUltra, 1000 (PEG1000) (81188- 250G, Sigma-Aldrich), *S. cerevisiae* strain Mav203 was from ProQuest™ Two-Hybrid system(PQ10001-01, Thermo Fisher Scientific), Deoxyribonucleic acid sodium salt from salmon testes (ss-DNA) (D1626-5G, Sigma-Aldrich), AccuTherm™ Microtube Shaking Incubator (I-4002-HCS, Labnet International, *Inc*.), JetStar™ 2.0 Endotoxin-free Megaprep Kit (232006, Genomed), and the plasmid DNA used in this study was Leucine rich repeat (LRR) and Malectin domain of TaRNR8 in pDEST32.

### Preparation of yeast competent cells

These transformation experiments were carried with Saccharomyces cerevisiae (haploid Mav203; MaV203 genotype is MATα; leu2-3,112; trp1-901; his3∆200; ade2-101; cyh2^R^; can1^R^; gal4∆; gal80∆; GAL1::lacZ; HIS3_UASGAL1_::HIS3@LYS2; SPAL10::URA3). An aqueous amino acid (10x AA mix) solution (1.35 g yeast synthetic drop-out medium supplements, 0.01795 g uracil, 0.1677 g histidine-HCl, 0.06535 g tryptophan in 100 mL ddwater) was prepared one day before yeast transformation and stored at 4 °C avoiding light. The 10x AA did not contain leucine due to the fact that successfully transformed yeast can synthesise leucine. All the transformation experiments were performed using the S.c. EasyComp™ Transformation kit (Thermo Fischer Scientific), unless stated otherwise. As an alternative we used a published transformation protocol (LiAc/ss-DNA/PE Gprotocol)^17^ modified as follows: addition of salmon sperm ss-DNA (final concentration was 0.2 mg/mL); a single colony of S. cerevisiae was cultured in 10 ml YPAD medium at 30 °C and 250 rpm overnight. When OD^600^ of the overnight culture reached between 3.0 and 5.0, the culture was diluted to an OD^600^ of 0.2 to 0.4 in a total volume of 10 ml of YPAD with 10x AA mix or without 10x AA mix. After the dilution, the cells were grown on at 30 °C and 250 rpm until OD^600^ reached 0.6 to 0.8. The cells were pelleted by centrifugation at 500 g for 5 minutes and the supernatant was discarded. The cells were then re-suspended in 10 ml of washing buffer (1M sorbitol, 10 mM Bicine-NaOH (pH = 8.35), 3% ethylene glycol, 5%DMSO, and water), followed by centrifugation at 500 g for 5 minutes again and carefully discarded the supernatant. The cell pellet was re-suspended once more in 1 ml Competence Solution (1 M sorbitol, 10 mM Bicine-NaOH (pH = 8.35) 3% ethylene glycol, 5%DMSO, 0.1 M LiAc, 1.25x AA mix). The competent cells were aliquoted (50 μl) into 1.5 ml sterile microcentrifuge tubes and frozen at −80 °C overnight.

### Yeast transformation

The competent cells were thawed at room temperature and then endotoxin-free plasmid DNA was added, followed by 500 μl of the Transformation Mix Solution (PEG1000 (36% w/v), 0.1 M LiAc, 0.2 mg/ml ss-DNA, 0.2 M Bicine-NaOH (pH = 8.35)) with or without nutrient supplements (The final concentration of AA mix was 1.25x). The information of washing buffer, competence solution, and transformation mix is detailed in Table 1. The cell suspension was then mixed by flicking the tube. The yeast was then heat-shocked at 37 °C in an AccuTherm™ Microtube Shaking Incubator and shaken every 15 minutes at 1500 rpm for 5 seconds. After the heat-shock, 50 μl of the transformation reaction was plated on synthetic complete “drop out” leucine (Sc-Leu) plates. The plates were then incubated at 30 °C for 3 days. All the yeast transformation experiments were performed 5 times independently and the data analyses including RSM were performed by using R.

## Additional Information

**How to cite this article**: Yu, S.-C. *et al*. Nutrient supplements boost yeast transformation efficiency. *Sci. Rep.* **6**, 35738; doi: 10.1038/srep35738 (2016).

## Figures and Tables

**Table 1:** The ingredients and concentrations of washing buffer, competence solution, and transformation mix.

Washing Buffer		Competence solution		Transformation Mix	
2M Sorbitol	25 mL	2M Sorbitol	25 mL	PEG1000 (60% w/v)	10.8 mL
1M Bicine-NaoH (pH:8.35)	0.5 mL	1M Bicine-NaoH (pH:8.35)	0.5 mL	1M LiAc	1.8 mL
Ethylene glycol	1.5 mL	Ethylene glycol	1.5 mL	ss-DNA (2 mg/mL)	1.8 mL
DMSO	2.5 mL	DMSO	2.5 mL	Bicine-NaOH (pH:8.35)	3.6 mL
Water	20.5 mL	1M LiAc	5 mL		
		10x AA mix	6.25 mL		
		water	9.25 mL		
Total	50 mL		50 mL		18 mL

Of note, if AA mix is added to the transformation mix, 1 mL of 10x AA mix is added into 7 mL transformation mix so that the final level of AA mix in the transformation mix is 1.25x.
